# Rates of carbon monoxide elimination in males and females

**DOI:** 10.14814/phy2.12237

**Published:** 2014-12-11

**Authors:** Gerald S. Zavorsky, Janet Tesler, Joshua Rucker, Ludwik Fedorko, James Duffin, Joseph A. Fisher

**Affiliations:** 1 Department of Health and Sport Sciences, University of Louisville, Louisville, Kentucky; 2 Department of Physiology and Biophysics, University of Louisville, Louisville, Kentucky; 3 Department of Physiology, University of Toronto, Toronto, Ontario, Canada; 4 Department of Anesthesia, Mount Sinai Hospital, University of Toronto, Toronto, Ontario, Canada; 5 Department of Anesthesia, University Health Network, University of Toronto, Toronto, Ontario, Canada

**Keywords:** Alveolar ventilation, carboxyhemoglobin, gender differences

## Abstract

The purpose of this study was to verify the previously reported shorter half‐time of elimination (*t*_½_) of carbon monoxide (CO) in females compared to males. Seventeen healthy subjects (nine men) completed three sessions each, on separate days. For each session, subjects were exposed to CO to raise the carboxyhemoglobin percentage (COHb) to ~10%; then breathed in random order, either (a) 100% O_2_ at poikilocapnia (no CO_2_ added), or (b) hyperoxia while maintaining normocapnia using sequential gas delivery, or (c) voluntary hyperpnea at~4x the resting minute ventilation. We measured minute ventilation, hemoglobin concentration [Hb] and COHb at 5 min intervals. The half‐time of reduction of COHb (*t*_½_) was calculated from serial blood samples. The total hemoglobin mass (Hb_TOT_) was calculated from [Hb] and estimated blood volume from a nomogram based on gender, height, and weight. The *t*_½_ in the females was consistently shorter than in males in all protocols. This relationship was sustained even after controlling for alveolar ventilation (*P *<**0.05), with the largest differences in *t*_½_ between the genders occurring at low alveolar ventilation rates. However, when *t*_½_ was further normalized for Hb_TOT_, there was no significant difference in *t*_½_ between genders at alveolar ventilation rates between 4 and 40 L/min (*P *=**0.24). We conclude that alveolar ventilation and Hb_TOT_ are sufficient to account for a major difference in CO clearance between genders under resting (nonexercising) conditions.

## Abstract (precis)

The rate of removal of carbon monoxide from the blood is different between males and females, and this difference is largely due to the difference in total hemoglobin mass between the genders and alveolar ventilation. If the hemoglobin concentration is normalized between the genders, and alveolar ventilation is similar between them, this gender difference is eliminated, and the carboxyhemoglobin decay is roughly equal between the genders.

## Introduction

Carbon monoxide (CO) poisoning is the most common cause of poisoning morbidity and mortality in the industrialized world (Ernst and Zibrak 1998). Unintentional CO exposure estimated 15,000 visits to the emergency departments each year in the United States, resulting in about 500 unintentional deaths (Centers for Disease Control and Prevention 2007). This likely underestimates the true incident of CO poisoning, as it does not include the many patients who visit clinics or family doctors, or those that are mis‐ or undiagnosed.

The recommended treatment for severe CO poisoning is hyperbaric oxygen (Weaver 2014), although its benefit in reducing adverse neurologic outcomes is questionable (Buckley et al. 2011). Nonetheless, such treatment facilities are seldom available at the site of first echelon of care. The decision of how to trade off the need for rapid initiation of treatment against optimizing its efficacy must take into account the severity of the poisoning, the rate of elimination of CO that can be effected at the first echelon of care.

The half‐time for CO elimination (*t*_½_) is generally affected by inspired oxygen partial pressure (P_I_O_2_) and minute ventilation (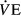) (Takeuchi et al. 2000; Zavorsky et al. 2012). Increases in 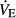 reduce arterial Pco_2_ (PaCO_2_) and cerebral blood flow, resulting in a reduction in brain O_2_ delivery (Rucker et al. 2002). Nevertheless, even with isocapnic hyperpnea at very high 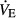 [see Fisher and colleagues for review (Fisher et al. 2011)], CO elimination can become perfusion limited at rest (Ishida et al. 2007), but reaches greater levels if 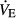 is accompanied by increases in cardiac output, as occurs with exercise (Zavorsky et al. 2012).

It has been postulated that another factor affecting *t*_½_ is gender. In 1950, while studying the effect of Po_2_ on *t*_½_ Pace et al. (1950) reported a significant difference in *t*_½_ with hyperoxia between males and females of 47 versus 36 min respectively. Although some investigators have confirmed this notion (Rode et al. 1972; Deller et al. 1992), others have refuted it (Burney et al. 1982; Weaver et al. 2000). Nevertheless, if a gender‐related difference exists, the explanation for this difference is uncertain. Mathematical models of CO uptake and elimination such as the Coburn, Forster, and Kane (CFK) equation (Coburn et al. 1965) do not have gender as a factor but some factors in the equation such as alveolar ventilation (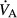), blood volume, and hemoglobin concentration [Hb] and pulmonary diffusing capacity for carbon monoxide (D_L_CO) differ between genders and may account for the differences in *t*_½_, but this too has not been investigated.

Consequently, the purpose of this study was to verify if the previously reported shorter *t*_½_ CO in females compared to males. We hypothesized that, in normal subjects, the effect of 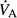 and total hemoglobin mass is sufficient to explain the observed gender difference in *t*_½_.

## Methods

Following institutional approval, nine females and nine males gave informed written consent to participate in the study. All subjects were healthy, nonsmokers, and with normal pulmonary function test results as reported by the clinical Pulmonary Function Laboratory at Mount Sinai Hospital, Toronto Canada.

Female volunteers were in the first 2 weeks of their menstrual cycle to ensure that they were in the estrogenic phase while participating in the study. This ruled out the possibility of pregnancy in the volunteers, and minimized the effects of progesterone, which include increased ventilation and increased endogenous CO production (Longo 1977).

**Figure 1.**
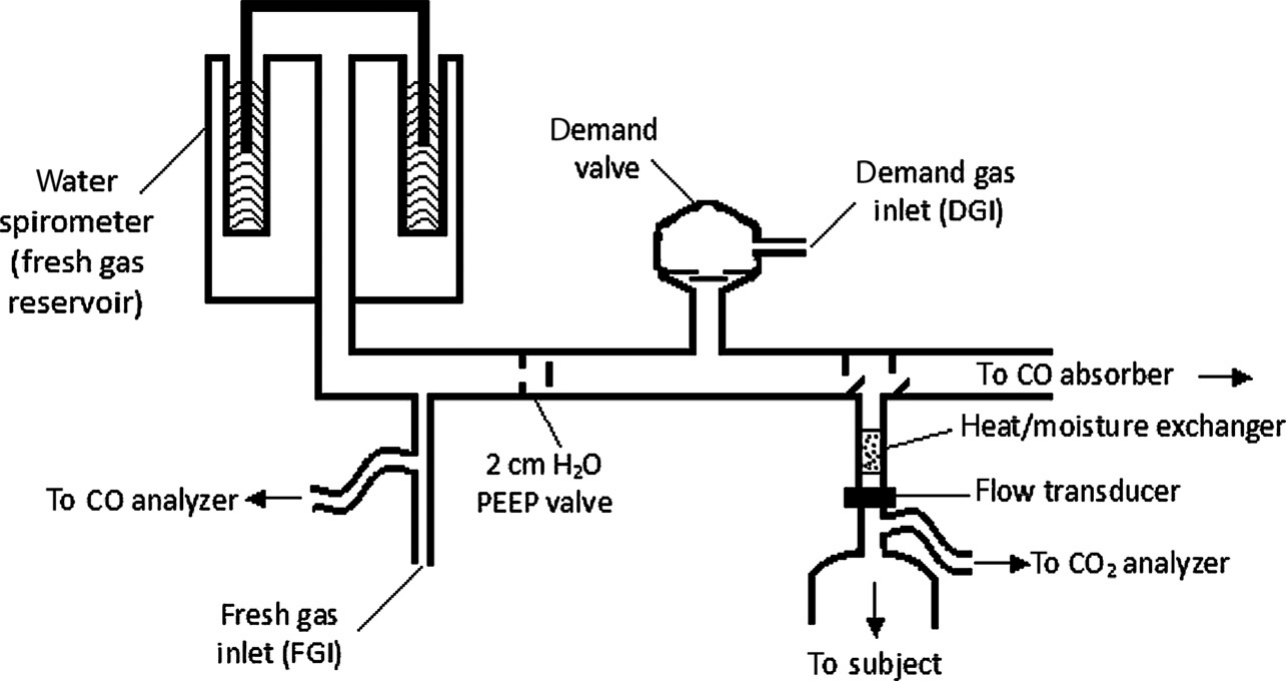
Breathing circuit. Fresh gas consisting of air or 100% oxygen is supplied through the fresh gas inlet (FGI) at a rate equal to the subject's resting ventilation and flows into the spirometer, from which the subject breathes. If ventilation increases, the reservoir empties during the course of the breath and the remainder of the breath is drawn through the demand regulator. Normocapnia is maintained when 6% carbon dioxide is supplied through the demand gas inlet (DGI).

The breathing circuit described by (Sommer et al. (1998) was used, with minor modifications (See Fig.****1). In brief, the circuit has two gas sources. The first source provides a constant flow. The second gas source provides flow through a demand regulator, and makes up the difference between the constant flow and 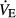 .

The study consisted of three sessions, A, B, and C. Each volunteer performed all three sessions and each session was performed on a separate day. The order of these sessions was randomized. Prior to the first session, general anthropometric data were collected. In addition, a venous blood sample was taken to determine hemoglobin concentration ([Hb]) (Cell‐Dyn 3500, Abbott, Mississauga, ON). The protocol is summarized in Table****1.

**Table 1.** Summary of the protocol

Section	Session	Gas entering the fresh gas inlet	Gas entering the demand gas inlet
Control 1	A, B, and C	Air	–
Exposure	A, B, and C	N/A	N/A
Elimination	A	O_2_	O_2_
	B	O_2_	6% CO_2_ bal O_2_
	C	O_2 _+ 6% CO_2_ bal O_2_	–

N/A, not applicable; bal, balance.

At the start of each session, the subject was seated comfortably, and a 20 gauge, 30 mm indwelling catheter was inserted into a forearm vein and attached to an intravenous extension and three‐way stopcock (for drawing blood samples). The subject was connected to the breathing circuit using a full face mask with a silicon seal (8930 series, adult medium, Hans Rudolph, Kansas City, MO). Systolic and diastolic blood pressures, and heart rate were recorded every 5 min (Datex AS/3, Helsinki, Finland). The analog signals for end‐tidal Pco_2_ (P_ET_CO_2_) (Datex Capnomac Ultima, Helsinki, Finland), inspired CO (P.K. Morgan Ltd., Chatham, Kent, England) were digitized and recorded on a commercial data acquisition system (Windaq, DATAQ Instruments Inc., Akron OH). Expiratory flow was measured using a fast response unidirectional flow transducer (SC520, VacuMed, and Ventura, CA). The digital output from the flow transducer was integrated to calculate 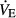 .

### Control tests

Prior to each exposure to CO, the volunteers breathed air for 10–20 min to allow acclimation to the breathing circuit. Once a steady state was reached (i.e. P_ET_CO_2_ remained constant), the subject breathed air for an additional 5 min during which baseline measurements of 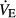, P_ET_CO_2_, heart rate, blood pressure were recorded.

### Exposure to CO

Subjects were exposed to ~1000 (range 900–1300) ppm CO in air from a reservoir through a non‐rebreathing valve. Venous blood was drawn every 5 min (0.5 mL) and analyzed for COHb (OSM3, Radiometer, Copenhagen, Denmark). CO exposure was discontinued when [COHb] was 10–12%. Required duration of CO exposure was 30–60 min.

### Elimination of CO

One of three methods of eliminating COHb was applied for 1 h. During session A, *poikilocapnic hyperoxia* (no CO_2_ added, P_ET_CO_2_ or PaCO_2_ allowed to vary freely), the constant gas flow consisted of O_2_ at a flow less than 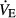 and the balance was made up of O_2_ supplied via the demand regulator. For session B, *normocapnic hyperoxia*, the constant O_2_ flow was set equal to the 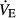 measured when breathing room air at rest, and the balance of 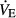 was supplied as 6% CO_2_, balance O_2_ via the demand regulator. The constant flow of O_2_ contributed to the elimination of metabolically produced CO_2_ and the gas from the demand regulator maintained hyperoxia but did not contribute to CO_2_ elimination. Normocapnia was maintained regardless of 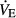 (and the volume of 6% CO_2_ balance O_2_ drawn through the demand regulator). Hyperoxia is a respiratory stimulant (Becker et al. 1996) resulting in an increase in 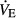 . When the gas from the demand regulator is O_2_, the PaCO_2_ is reduced (poikilocapnia), limiting the increase in 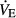 . When the gas provided through the demand regulator is 6% CO_2_ balance O_2_, PaCO_2_ will remain at baseline (normocapnia) and 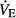 will double or triple (Becker et al. 1996). In session C, isocapnic hyperpnia (IH), the O_2_ flow was equal to that of air at rest (to provide the gradient for the elimination of metabolic CO_2_ production and normocapnia) with balance flow of 6% CO_2_ in O_2_ to maintain the mormocapnia. The flow of the 6% CO_2_ bal O_2_ was set to three times the O_2_ flow; the total flow into the spirometer was then baseline 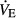 , plus three times baseline 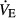 , totaling four times baseline 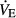 . The subjects were instructed to breathe from the reservoir (using any pattern of breathing) so that they maintained a constant level (height) of the spirometer drum. This ensured that subjects maintained 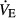 equal to four times resting 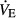 throughout session C. Subjects were blinded between session A and session B as both protocols entailed drawing gas from the demand regulator.

### Analysis

P_ET_CO_2_ was obtained from the Pco_2_ tracing using the peak detector function of the data acquisition software. After manually removing artificially low and “double peaks” from the tracing, remaining peak values were imported into Microsoft Excel where the average values for each section (control, exposure, and elimination) of each session (A, B, and C) were determined.

### Calculations

*t*_*½*_*:* COHb were plotted versus time and fitted with an exponential curve of the form:
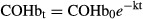


Where COHb_0_ and COHb_t_ are the COHb concentrations at time 0 and time t, respectively, and k is the rate constant. The rate of elimination of COHb can be stated in terms of the half‐life of elimination of COHb *t*_½_, which is related to the rate constant k, by the following equation:
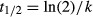


Hb_TOT_*:* Hb_TOT_ is the product of circulatory blood volume and [Hb]. Circulatory blood volume (in liters) was calculated from the following equations (Hidalgo and Nadler 1962):
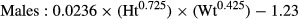

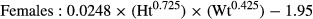
where Ht and Wt are height in cm and weight in kg respectively.


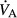
 : 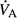 was calculated by subtracting dead space ventilation from minute ventilation. Dead space was considered to be the sum of the circuit dead space (170 mL) and the anatomical dead space (the subject's weight in pounds, in mL). Dead space ventilation is the product of dead space volume and breathing frequency.

### Statistical analysis

For each subject, the *t*_½_ determined for each session were plotted against the inverse of the subject's average 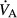 (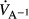 ) during that session. Since the relationship between *t*_½_ and 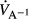 is linear (Coburn et al. 1965), a least squares regression equation was fitted for *t*_½_ versus 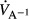 for each person to determine each individual's slope and intercept. A one‐way analysis of variance (ANOVA) was used to determine the effect of gender on these slopes and intercepts. This analysis was repeated with a two‐way ANOVA which included both Hb_TOT_ and gender. A two‐way ANOVA was used to determine the effects of the session performed (A, B, or C); and the gender of the subject on the particular parameter (inspired [CO], time, [COHb]). Tukey's test was used for post hoc testing (if required). A two‐way ANOVA, blocking for subject, was used to determine the effect of the section (control, exposure to CO, or elimination of CO) and gender of the subjects on these parameters for sessions A, B, and C. If there was a significant interaction between ‘section’ and ‘gender’, then the data were reanalyzed separately for males and females. Tukey's test was used for post hoc testing (if required). All other statistical analyses were performed using unpaired t‐tests. Results were considered significant at *P* < 0.05.

## Results

One female subject did not complete all three sessions and therefore her data are not included. Table****2 provides anthropometric data for the participating subjects. All parameters differed significantly between males and females (*P *<**0.05) except for age.

**Table 2.** Anthropometric data

Gender	Age (years)	Weight (kg)	Height (cm)	FRC (L)	TLC (L)	D_L_CO (mL/min/ mmHg)	[Hb] (g/dL)
Females (*n* = 8)	29 (11)	59 (6)	162 (5)	2.7 (0.5)	5.4 (0.6)	23.8 (1.7)	12.3 (0.9)
Males (*n* = 9)	27 (11)	73 (11)*	177 (6)*	3.5 (0.6)*	6.9 (0.9)*	33.5 (3.0)*	14.1 (0.5)*

Mean (SD).

* *P* < 0.05 compared to females; FRC, functional residual capacity; TLC, total lung capacity; D_L_CO is pulmonary diffusing capacity for CO from the single breath‐hold maneuver; [Hb], hemoglobin concentration.

There was no significant difference between sessions or between the genders for average inspired CO concentration (~1100 ppm CO), peak COHb (~10%), or exposure duration (31 min). Therefore, exposure conditions were the same for males and females and between sessions (Table****3).

**Table 3.** CO exposure parameters during the three sessions

Session	Males	Females	Both
Session A			
Average inspired [CO] (ppm)	1104 (84)	1044 (71)	1078 (81)
Exposure Duration (min)	33.7 (6.0)	30.6 (6.2)	32.0 (6.0)
Peak COHb (%)	10.3 (0.4)	10.5 (0.5)	10.3 (0.5)
Session B			
Average inspired [CO] (ppm)	1106 (49)	1135 (120)	1129 (94)
Exposure Duration (min)	31.0 (3.9)	27.9 (5.2)	29.5 (4.6)
Peak COHb (%)	10.3 (0.4)	10.5 (0.6)	10.4 (0.5)
Session C			
Average inspired [CO] (ppm)	1128 (45)	1072 (89)	1099 (72)
Exposure Duration (min)	31.3 (3.6)	29.4 (6.6)	30.2 (5.1)
Peak COHb (%)	10.4 (0.3)	10.5 (0.8)	10.4 (0.6)

Mean (SD).

**Figure 2.**
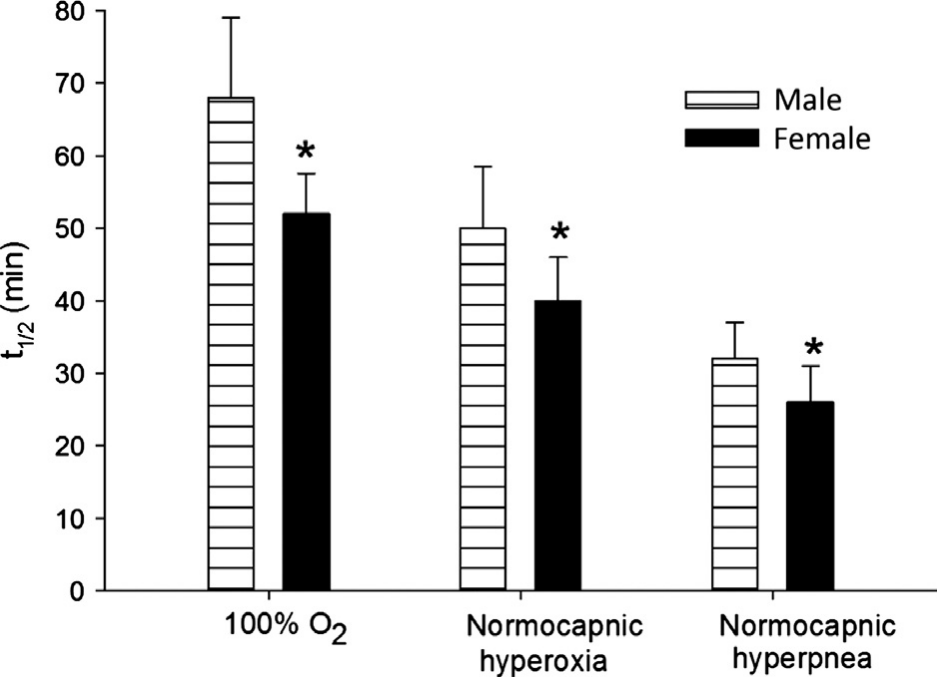
Half‐life of carboxyhemoglobin (*t*_1/2_) between males and females under three different conditions. (1) poikilocapnic hyperoxia; (2) normocapnic hyperoxia; (3) Normocapnic hyperpnea: 4x resting expired ventilation rate.

**Figure 3.**
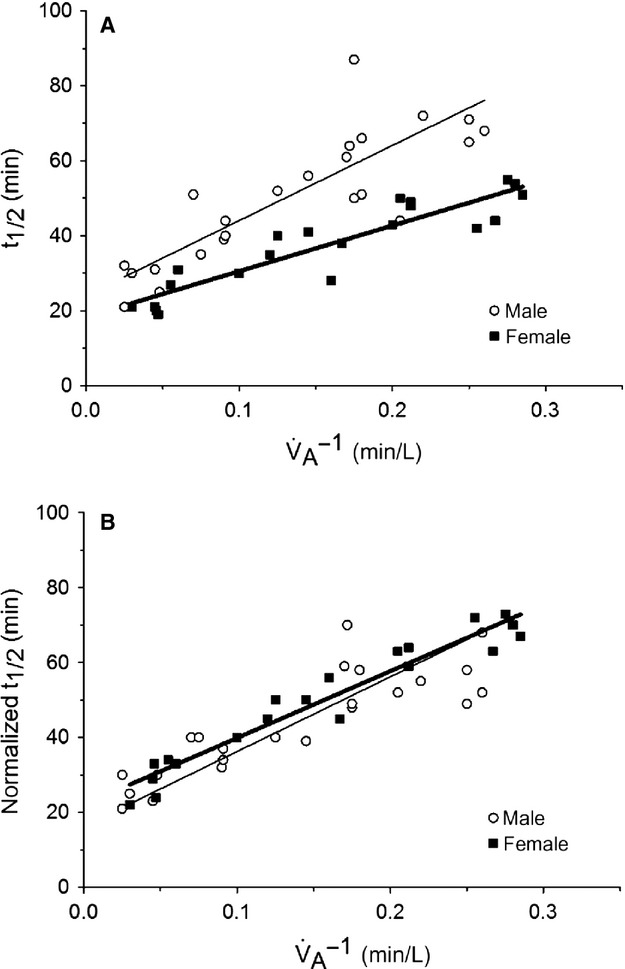
(A) The relation between carboxyhemoglobin half‐life and the inverse of the alveolar ventilation rate, not normalized for the total hemoglobin mass. (B) The relation between carboxyhemoglobin half‐life, normalized for the total hemoglobin mass and the inverse of the alveolar ventilation rate. The gender difference in carboxyhemoglobin half‐life is eliminated when normalizing to total hemoglobin mass.

When the *t*_½_ between males and females were compared for each session, the female *t*_½_ was always significantly less than the male *t*_½_ (*P *<**0.05; see Fig.****2). In a model using the slope of *t*_½_ versus the inverse of alveolar ventilation (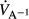) as the outcome variable and gender as the predictor, the coefficient for gender is statistically significant (*P *=**0.002). With intercept as the outcome, the gender coefficient is not statistically significant indicating that gender would not affect the *t*_½_ at infinite 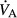 (i.e. the y‐intercept). When gender *and* Hb_TOT_ were included as predictors of the slope and the intercept in a two‐way ANOVA, the coefficient for gender remained insignificant for the intercept (*P *=**0.080) and was no longer statistically significant for the slope (*P *=**0.235) (Fig. 3A and B).

All cardiovascular and ventilatory parameters were compared to control (breathing air) from that day's session (Table****4). Male P_ET_CO_2_ was 9% higher than female P_ET_CO_2_ for all sessions. However, Pco_2_ has no known effect on the rate of CO elimination and therefore cannot be expected to contribute to the difference in *t*_½_ between males and females.

**Table 4.** Cardiovascular and ventilatory parameters during sessions A,B, C

Parameters	Session	Males	Females
Session A			
HR	Control	66 (11)	66 (9)
mBP		92 (5)	87 (8)
RPP		6080 (1130)	5760 (1070)
*V*_A_		4.0 (0.4)	4.3 (1.8)
P_ET_CO_2_		39.9 (2.9)	36.4 (2.4)
HR	Exposure	67 (11)	65 (5)
mBP		91 (6)	88 (6)
RPP		6060 (1070)	5690 (560)
*V*_A_		4.1 (0.7)	3.3 (0.8)
P_ET_CO_2_		39.6 (3.3)	36.5 (2.6)
HR	Elimination	62 (11)*	63 (4)*
mBP		92 (5)	90 (7)
RPP		5710 (1090)	5680 (690)
*V*_A_		4.7 (0.8)**	4.4 (1.6)
P_ET_CO_2_		36.6 (3.6)*	32.4 (1.2)*
Session B			
HR	Control	67 (11)	64 (8)
mBP		89 (4)	88 (9)
RPP		5920 (935)	5700 (1100)
*V*_A_		5.0 (1.0)	3.4 (1.0)
P_ET_CO_2_		41.6 (2.7)	38.1 (3.6)
HR	Exposure	68 (11)	66 (8)
mBP		87 (5)	87 (11)
RPP		5920 (1030)	5810 (1210)
*V*_A_		4.8 (1.2)	3.6 (0.7)
P_ET_CO_2_		41.5 (2.4)	37.8 (4.0)
HR	Elimination	63 (11)**	65 (8)
mBP		91(5)	88 (9)
RPP		5710 (1090)	5740 (1060)
*V*_A_		8.6 (3.0)*	6.0 (1.6)*
P_ET_CO_2_		40.6 (2.0)	38.1 (2.8)
Session C			
HR	Control	72 (12)	66 (9)
mBP		90 (9)	87 (8)
RPP		6520 (1290)	5700 (947)
*V*_A_		5.4 (1.5)	4.0 (2.4)
P_ET_CO_2_		41.0 (2.4)	36.9 (2.6)
HR	Exposure	70 (11)	65 (8)
mBP		89 (5)	86 (9)
RPP		6240 (1070)	5660 (1010)
*V*_A_		4.9 (1.2)	3.6 (1.9)
P_ET_CO_2_		39.9 (3.1)	36.1 (5.3)
HR	Elimination	68 (9)	68 (9)
mBP		95 (5)*	92 (10)*
RPP		6450 (950)	6320 (1210)
*V*_A_		27.7 (8.1)*	21.0 (4.4)
P_ET_CO_2_		40.9 (1.9)	38.4 (2.1)

Mean (SD).

* Significantly different from control (male and female data grouped, *P* < 0.05).

** Significantly different from control (*P* < 0.05); end tidal CO_2_ concentration was significantly higher for the males compared to the females during all three sessions. HR, heart rate (beats/min); mBP, mean blood pressure (mmHg); RPP, rate‐pressure product; *V*_A_, alveolar ventilation (L/min); P_ET_CO_2_, partial pressure of end‐tidal CO_2_ (mmHg).

## Discussion

The main finding of this study is that females had a shorter CO elimination *t*_½_ compared to males, even when controlling for ventilation rates. This difference was magnified at lower 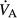
‘s. For example, when 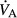 was ~4 L/min, the *t*_½_ was ~28 min longer in men compared to women (Fig. 3A). This makes sense because *t*_½_ represents the steep parts of their hyperbolic curves at low 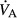
‘s and the underlying mass of CO will have a big difference on *t*_½_. However, when 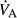 was ~40 L/min, *t*_½_ was only 9 min longer in males compared to females (Fig. 3A). Again, this makes sense since the greater the 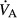 , the less the effect of total body Hb. Thus, a ~10‐fold increase in 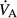 reduces the gender difference of *t*_½_ by about threefold when 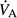 is increased from ~4 to ~40L/min.

As the gender difference in *t*_½_ disappears when normalizing for Hb_TOT_ it suggests that the difference in *t*_½_ is mainly due to the difference in CO stores. Several previous studies have reported *t*_½_ in males and females (Pace et al. 1950; Rode et al. 1972; Burney et al. 1982; Deller et al. 1992; Weaver et al. 2000). Pace et al. (1950) were the first to note a gender‐related difference in *t*_½_. These authors did not offer an explanation for the gender‐related difference. Furthermore, no additional information (height, weight, [Hb], 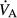) were provided for their subjects, so we were unable to determine whether differences in 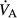 and Hb_TOT_ could have explained their findings.

Rode et al. (1972) observed a gender‐related difference in *t*_½_ of 3.7 h and 2.5 h for 336 males and 265 females breathing air. However, they determined the *t*_½_ in an unusual manner. They measured each of their subject's COHb once and plotted this against the log of the time since their last cigarette (as reported by the subject). They then plotted a line of best fit to both the female and male data to obtain the *t*_½_. This method of calculating *t*_½_ has a poor precision as it pools COHb measurements from a heterogeneous group of subjects instead of calculating each individual subject's *t*_½_. Furthermore, their population consisted of long‐term smokers chronically exposed to low levels of CO. Consequently, their findings may not be applicable to healthy nonsmokers (Weaver et al. 2000), and their observed gender‐related difference must be interpreted with caution. These authors also attributed the difference in *t*_½_ between the genders to the difference in total blood volume. However, they did not test this hypothesis.

Deller et al. (1992) also examined *t*_½_ in male and female smokers. Breathing room air, female *t*_½_ (3.2 ± 0.4 h, *n* = 7) was significantly shorter (*P* < 0.01) than male *t*_½_ (4.5 ± 1.1 h, *n* = 6). They attributed the gender difference due to the fact that females have less muscle mass, and therefore less myoglobin mass, than males. However, while males may have more myoglobin mass than females, about 10–15% of total body CO is bound to myoglobin in skeletal and cardiac muscle, while the rest is chemically bound to Hb (Coburn and Forman 1987). Thus, Deller and colleagues did not consider a difference in HbTOT between males and females.

Two retrospective studies of CO‐poisoning found no difference in the rates of CO elimination between males and females. Burney et al. (1982) determined *t*_½_ for 33 victims of CO poisoning treated with 100% O_2_ to be 137 min. While they did not report the proportion of male or female patients, they stated that gender did not affect *t*_½_. Weaver et al. (2000) also performed a retrospective chart review of CO‐poisoning victims from 1985 to 1995 in the Salt Lake City area. In their step‐wise multiple linear regression analysis of 93 patients, they found that the only independent variable influencing *t*_½_ was arterial Po_2_ and that male and female *t*_½_ did not differ. However, they admit that *t*_½_ determined from retrospective observational studies of CO poisoning often differ from those obtained experimentally.

In retrospective studies, investigators cannot control the nature and duration of exposure, nor the speed with which treatment was administered and the nature of that treatment. In the study by Burney and colleagues, the victims had been exposed to the same source of CO, but the time of exposure ranged from approximately 40 to 170 min (Burney et al. 1982). All victims were treated with high‐flow O_2_ administered by “rebreather mask” (a valveless face mask designed to store the gas from the anatomical dead space during exhalation and provide it as the entrained gas during inhalation), but victims were treated at different hospitals. In the study by Weaver et al. (2000), the victims were exposed to unknown levels of CO for unknown periods. All the victims were treated with 100% O_2_ via either a non‐rebreathing face mask or an endotracheal tube. The fraction of inspired O_2_ (F_I_O_2_) delivered by a face mask depends on the patient's breathing frequency and pattern, the fit of the mask and the O_2_ flow (Wexler et al. 1975). For example, 100% O_2_ delivered at 15 L/min through a standard hospital face mask results in a true F_I_O_2_ of 0.70 ± 0.10. Even at 30 L/min, F_I_O_2_ is 0.85 ± 0.05 (Wexler et al. 1975). None of these confounding variables was standardized or reported in either study. As a result any gender‐related differences in *t*_½_ might have been masked by variations in F_I_O_2_ and/or differences in 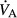 . Indeed, Weaver et al. (2000) acknowledged that differences in 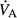 and F_I_O_2_ could have contributed to the large SD of their average *t*_½_.

### Mathematical modeling

Although numerous mathematical models have been developed to predict the uptake and/or elimination of CO none has been shown to adequately address the gender‐related difference in *t*_½_ discovered by Pace et al. (1950). Indeed many of these models were only intended to predict CO uptake, rather than elimination (Forbes et al. 1945; Lilienthal and Pine 1946; Pace et al. 1946; Hatch 1952; Ott and Mage 1978; Chung 1988). Of the remaining models, four were tested against data from males only (Peterson and Stewart 1970; Tyuma et al. 1981; Singh et al. 1991; Selvakumar et al. 1993), two were tested in animals only (Wagner et al. 1975; Halebian et al. 1984), one was tested in only two subjects (Goldsmith et al. 1963), and one was tested against another model (Benignus 1995).

The CFK equation was developed in 1965 by Coburn et al. (1965) based on theoretical assumptions and was originally validated using data from three healthy males and from three anemic patients with increased rates of endogenous CO production (sex undisclosed). The peak COHb measured was 2.5%, which is well below toxic levels, as well as the COHb levels of the subjects of Pace et al. (1950). It remains unknown if any of the subjects of Coburn et al. (1965) were female.

Only the CFK equation has been tested against data recorded from female subjects (Peterson and Stewart 1975; Joumard et al. 1981). In 1975, Peterson and Stewart attempted to validate the CFK equation in both males and females (Peterson and Stewart 1975). Over 12 experiments, they exposed 22 subjects (three female) to various levels of CO at various levels of exertion and periodically measured [COHb]. In five of these experiments, they measured COHb during elimination as well. Of their 429 measurements of COHb only 34–40 were collected during elimination. Using the CFK equation they predicted these 429 COHb and compared these predictions with the measured values. They obtained equally good correlations between the predicted and measured values for both the male and female subjects and concluded that the CFK equation predicts uptake and elimination equally well for males and females. However, they did not perform separate analyses on COHb values obtained during exposure and during elimination. As a result, even had there been a significant difference between the males and females during elimination, this difference would have been masked by the numerous measurements taken during exposure. Furthermore, it is unknown if any of the female subjects performed any of the five experiments involving elimination.

Similarly, in 1981, Joumard and colleagues also attempted to validate the CFK equation (Joumard et al. 1981). They measured COHb levels in 37 males and 36 females before and after 2 h exposures to the CO‐polluted atmosphere (mean [CO] = 14 ppm). Based on continuous measurements of atmospheric [CO] they predicted the postexposure COHb levels using the CFK equation. They found that the equation predicted well for both men and women. However, as in the original experiment of Coburn et al. (1965), the measured COHb levels were very low (2%) and as for Peterson and Stewart (1975), they did not separate periods of CO uptake from periods of elimination. Therefore, one cannot conclude from any of the above studies that the CFK equation predicts *t*_½_ equally well for women and men. Consequently, we suggest that the basis for the findings of Pace et al. (1950) remained unknown until the present study.

### Limitations

First, subjects were exposed to a COHb level of only 10–12%. At steady state, the CO distribution between the blood and tissues are similar for COHb between 0 and 60% (Coburn 1970). At higher [COHb], myoglobin may bind more CO due to the relative hypoxia in the myocytes (Coburn and Forman 1987), and since males have more myoglobin, at high [COHb], they may have prolonged *t*_½_ due to increased myoglobin binding of CO. In addition, despite the prolonged rate of CO exposure in our subjects, no steady state was achieved (Longo and Hill 1977). The effect on the calculation to half‐time of elimination however, should be very nearly the same in both genders.

Second, total blood volume was calculated using the equations of Hidalgo and Nadler (1962), which were developed by least square regression analysis of height and weight data from 92 males and 63 females who had total blood volume calculated by the radioactive iodinated human serum albumin method; the total blood volume was not actually measured. Correlation coefficients for the male and female equations were 0.78 and 0.86, respectively (Hoffman et al. 2012), with standard deviations about 0.50 L for both male and female equations (Wexler et al. 1975). Nevertheless, measured total blood volume, may not have been much more accurate than calculated values. In 1950, Sterling and Gray (1950) described the chromium‐51 (Cr^51^) method of determining red cell volume (RCV; from which total blood volume can be determined) which, along with iodinated albumin, is considered the ‘gold standard’ for total blood volume measurement. They determined the accuracy of their total blood volume calculation in five humans (three male, two female) to be within 3% (stated as such since a 3% difference was the largest per cent difference of the five).

To ensure that our results were not greatly influenced by the choice of equations to determine total blood volume, we recalculated total blood volume with equations developed by Wennesland et al. (1959) (males) and Brown et al. (1962) (females). With these new total blood volume values, the findings did not change (i.e. the gender coefficient was not significant when Hb_TOT_ was in the model).

Third, our study did not take into account other factors that also differ between males and females, which could have affected CO elimination, D_L_CO, and myoglobin. D_L_CO was significantly greater in the males of this study. According to the CFK equation, with all other factors being equal, a higher D_L_CO should result in a shorter *t*_½_. However, the relationship between *t*_½_ and D_L_CO is not a simple one. Rearranging the CFK equation to solve for *t*_½_ as a function of D_L_CO predicts a hyperbolic curve, in which an increase in D_L_CO decreases *t*_½_. There is a vertical asymptote of 0 (suggesting an infinitely large *t*_½_ with a D_L_CO of 0); however, the horizontal asymptote is not 0, implying that other factors limit *t*_½_ at infinite D_L_CO. One of these factors is 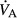 , because with increasing 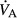 the horizontal asymptote approaches 0.

Total myoglobin body stores are considered a significant reservoir for CO with 10–15% of total body CO being bound to myoglobin (Coburn and Forman 1987). Males, by virtue of their greater muscle mass, have more myoglobin than females. Having a greater total myoglobin content is similar to having a greater Hb_TOT_; it increases total CO body stores (for a given [COHb]) and thus acts to prolong *t*_½_. The male subjects in this study weighed on average 20% more than the female subjects, and even if it is assumed that the male subjects had 50% more myoglobin than the female subjects, this factor represents only a 5–7% increase in their total body CO stores. Compared to the large difference in total body CO stores between males and females due to the differences in Hb_TOT_, we suggest that the effect of myoglobin is negligible. However, it cannot be certain that differences in myoglobin do not play a role and this factor warrants further investigation.

### Implications

This study indicates that the rate of clearance of CO influenced by gender only to the extent that the relationship of total body CO stores to 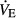 differ from that of males. These factors, not merely gender, must be taken into account when assessing clearance of CO. In particular, conditions that alter Hb_TOT_ and 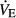 must be considered regardless of gender. For example, anemia will tend to shorten *t*_½_ whereas polycythemia (which is common in female smokers) will tend to lengthen it. Similarly, any condition which alters 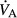 will also affect *t*_½_. For example, anxiety induced hyperventilation will lower *t*_½_ whereas hypoventilation due to emphysema, or a reduced level of consciousness will raise *t*_½_. Furthermore, limitations in cardiac output will affect *t*_½_ with any type of treatment (Ishida et al. 2007; Zavorsky et al. 2012). Thus, a prediction of *t*_½_ from standard tables not taking gender into account (i.e., most text books) will be erroneous. Less so if gender is taken into account, and even less so if cardiac output and ventilation are taken into account. Optimal predictions will require, in addition, a thorough clinical assessment.

## Acknowledgments

The authors would like to thank Kaleen Lavin and Allison Straub for the creation of the figures.

## Conflicts of Interest

JAF, JD, JR, JT, and LF are members of a team that subsequently applied principles similar to the apparatus used for this study to develop a device for clearance of CO from the lungs. The intellectual property for this device has been assigned to Thornhill Research Inc. (TRI), by the University Health Network where they are employed. These authors have shares in TRI and JAF and JD receive salary support from it. GZ has no conflict of interest to report.
